# Probiotic dairy products and consumption preferences in terms of sweetness sensitivity and the occurrence of childhood obesity

**DOI:** 10.3389/fpsyg.2022.980348

**Published:** 2022-09-30

**Authors:** Marek Kardas, Wiktoria Staśkiewicz, Ewa Niewiadomska, Agata Kiciak, Agnieszka Bielaszka, Edyta Fatyga

**Affiliations:** ^1^Department of Food Technology and Quality Evaluation, Faculty of Health Sciences in Bytom, Medical University of Silesia, Zabrze, Poland; ^2^Department of Biostatistics, Faculty of Health Sciences in Bytom, Medical University of Silesia, Bytom, Poland; ^3^Department of Preventive Medicine, Medical University of Silesia in Katowice, Bytom, Poland

**Keywords:** probiotics, dairy product, fermented food, yogurt, preferences, obesity

## Abstract

Fermented dairy products such as yogurt contain many bioactive compounds. In addition, probiotic yogurts are an invaluable source of probiotic bacteria and are a group of probiotic products best accepted by children. There is plenty of research indicating an interdependence between yogurt consumption, body mass index, and adipose tissue percentage, which suggests that yogurt consumption may contribute to reducing the risk of becoming overweight or obese. In turn, the occurrence of overweight and obesity may be accompanied by a reduced sensitivity to sweetness, which modifies food preference selection and acceptance, including with yogurt. This study aimed to assess the preferences and consumption of yogurt in terms of sensitivity to recognize sweetness and obesity in a group of 7–9-year-old children. Body mass index and adipose tissue percentage obesity indicators were determined, and the frequency of fermented milk product consumption was assessed about the results of the sweetness recognition test as well as yogurt preferences. There was no significant relationship between body weight and the frequency of fermented milk product consumption. Correlations were found between the values of body mass index and the ability to recognize sweetness, which was significantly better recognized by underweight children or at normal body weight, moreover, those children with a higher ability to recognize sweetness significantly more frequently preferred plain unsweetened yogurt.

## Introduction

Fermented foods have been consumed by humans for 1,000 years. Initially, they were most likely produced for their extended shelf life (Tamang et al., 2020). Currently, fermented foods are defined as “foods or beverages produced by the controlled microbial growth and enzymatic conversion of major and secondary food components” (Food and Agriculture Organization of the United Nations, 2011). Fermented dairy products hold a special place amongst many kinds of fermented foods (Savaiano and Hutkins, 2021). One of the most frequently consumed fermented dairy products is yogurt, which is widely accepted by consumers around the world for its high nutritional value, unique taste, and health benefits (Tremblay and Panahi, 2017; Zhang et al., 2020). According to data from Statistics Poland, in 2019 average monthly yogurt consumption in Poland was 0.54 liters per person and an upward trend has been observed (Central Statistical Office (CSO), n.d.). In the United States, yogurt production increased by more than 4% between 1995 and 2019, and yogurt consumption over the course of the last decade steadily increased (Cifelli et al., 2020). Yogurt is considered a healthy food choice due to its high digestibility and the bioavailability of its nutrients (Gómez-Gallego et al., 2018; Baspinar and Güldaş, 2020). The potential health benefits of yogurt, however, may be reduced depending on the add-ins included and the degree of processing that takes place (Sayon-Orea et al., 2017). Adding ingredients to yogurt apart from fruit or whole-grain cereals can increase sugar and total fat amounts, making it less nutritionally valuable than plain unsweetened yogurt (An and Jiang, 2017). Probiotic yogurt, which contains strains of probiotic bacteria in strictly defined amounts, is the most frequently used carrier for the delivery of probiotic strains of microorganisms widely accepted to be safe and healthy (Ali et al., 2020; Nyanzi et al., 2021). Current recommendations for the consumption of dairy products vary by region. Most national recommendations for dairy consumption call for 3–4 servings/cups/portions per day (Weaver, 2014). According to the Polish Pyramid of Healthy Nutrition and Lifestyle for Children and Youth, 3–4 glasses of milk should be consumed daily during this period of development, which can be replaced with plain unsweetened yogurt, kefir, buttermilk, and some types of cheese (National Center for Nutrition Education (NCNE), n.d.). Whereas, Dietary Guidelines for Americans (GDA) from 2020 to 2025 recommends that American children, depending on their age, consume 2–3 servings (converted into cups) of low-fat or non-fat milk, buttermilk, yogurt, kefir, frozen yogurt, dairy desserts, cheese and their soybean alternatives (Hess et al., 2020; USDA, 2020). Most often, children prefer strawberry, raspberry, forest fruit, vanilla, and chocolate yogurts (Stankiewicz and Lange, 2012; Kamber and Harmankaya, 2019). By adding fruit to yogurt, its flavor and some nutritional values such as vitamin C and minerals are improved, and the variety of products available is increased. If the product containing fruit is not sweetened and is produced with no or minimal additives, it is as healthy as plain unsweetened yogurt (Voşgan et al., 2016). Overweight and obesity are global public health problems. According to the World Health Organization (WHO), overweight and obesity are defined as excessive or abnormal fat accumulation that may affect human health (Sayon-Orea et al., 2017). According to WHO data, in 2025, 167 million people – adults and children were either overweight or obese (World Health Organization (WHO), n.d.). More and more studies confirm the correlation between the consumption of milk products and a reduced risk of overweight and obesity (World Health Organization, 2015; O'Sullivan et al., 2020). Consumer research shows that one of the most important factors in determining food choice is taste (Mennella et al., 2014). Due to this important role in nutrition, the relationship between taste preferences and the prevalence of overweight and obesity in children is highly relevant. Moreover, eating habits develop in early childhood and remain stable throughout adolescence and adulthood, thus having an impact on health both in the short term and in the long term (Lanfer et al., 2013; Divert et al., 2017). Considering the impact of dairy product consumption and taste preferences, an important element of which is the ability to recognize and accept sweet taste on the risk of overweight and obesity, the current study was implemented. The study aimed to determine the relationship between the weight of the subjects and the frequency of consumption of fermented dairy products, to examine the correlation between body mass index values and sweet taste recognition, to determine the relationship between the frequency of consumption of fermented dairy products and the result of the sweet taste identification test and its potential impact on yogurt selection preferences.

## Materials and methods

The stages of research included anthropometric measurements (BMI body mass index and FATP adipose tissue percentage), assessment of the frequency of consumption of a fermented dairy product, a test of the ability to recognize sweets, and analysis of yogurt selection preferences.

### Trial study

Research results were collected as part of an educational project addressed to second-grade primary school children (contract WND-POWR.03.01.00-00-C036/16 from the National Center for Research and Development).

The research was conducted with the consent of the Bioethics Committee of the Medical University of Silesia in Katowice. Within the study, in each of the 10 counties of the Upper Silesian Agglomeration, a simple random selection of one class of 7–9-year-old children from a random primary school was made. With these assumptions: total population size *N* = 250, estimated fraction 50%, confidence level 95%, the margin of error 3%, and the estimated sample size was 203. The final study group consisted of 200 s-grade children aged 7–9, but complete research material was collected from 160 out of 200 pupils (return factor 80%). The research covered a total of six schools and 9 s-grade classes. Ultimately, complete research material was collected from 160 students, including 70 (43.8%) girls and 90 (56.3%) boys.

### Inclusion and exclusion criteria from the study

The criterion for inclusion of students in the research was active participation in classes at the lower primary level (grade II) and the appropriate consent to participate in the research by their parents or legal guardians. The criterion for exclusion from the study was lack of consent of parental or legal guardian consent to participate or if some stages of participation were incomplete.

### Anthropometric measurements: Body mass composition analysis

#### BMI (kg/m^2^)

Measurements of height and weight were carried out in the school nurse’s offices using a medical scale with a stadiometer (WPT 60/150 OW model). The results were the basis for the assessment of the height and weight ratio about the standards for the Polish population and the WHO recommendations regarding height, weight, and BMI.

BMI was determined on the basis of the formula: BMI = (body weight [kg]/height [m])^2^.

Crude BMI scores were analyzed in separate age categories from 7 to 9 years. A general analysis of body mass index was performed by determining standardized BMI values based on the LMS (Least Mean Square) method presented by the World Health Organization (n.d.). The cut-off values for standardized BMI values were adopted in accordance with the assumptions of the World Health Organization (n.d.). Group comparative analyses were based on standardized values or BMI categories (underweight, norm, overweight, obesity).

#### FATP (%)

The percentage of adipose tissue FATP (%) was assessed using the bioelectric impedance analysis (BIA) method, using the TANITA BC 418 MA device, which is equipped with a system of eight polar measuring electrodes and is 93/42 EEC certified.

The tests were carried out according to the manufacturer’s recommendations. Because the results of the study may be affected by hydration status, avoiding strenuous exercise and drinking fluids were recommended to participants in preparation for measurements.

The cut-off points specified in the Tanita operating instructions were applied. Group comparative analyses were based on raw values or FATP categories (underweight, norm, overweight, obesity).

### Assessment of fermented milk product consumption frequency

The assessment of the frequency of fermented dairy product consumption from the month before the study was carried out using a questionnaire made for this study. It contained a single-choice question regarding the frequency of consumption of this type of product by children and questions about their BioData, i.e., personal information such as age and sex. The questionnaires were completed by the parents or legal guardians of the students at school meetings after providing oral instructions on how to complete them.

### Test of the ability to recognize sweetness among the four basic flavors

As part of the research, the ability to recognize basic tastes, including sweetness, was assessed.

The assessment of the ability to recognize sweetness was carried out in conditions meeting ISO 8589 requirements (PN-EN ISO, 2007), i.e., in a sensory laboratory equipped with six individual stands separated by partitions and connected by a window with a sample preparation room. Due to the age of the evaluators, the sensory tests were carried out with the direct participation of the assigned tutor in a “guardian-student” mode, and the procedures used in the sensory evaluation were adjusted to the level of the children.

Standard solutions of basic flavors were prepared by the requirements of ISO 3972 (PN-ISO 3972:2016-07/AC1, 2016) based on demineralized water at the concentrations given in Table 1. Reagents dedicated to sensory tests of prod. Sigma-Aldrich, United States (Table 1).

**Table 1:** Concentrations of the solutions used in the taste recognition test.

Taste	Reference substance	CAS registry number	Concentration of solution (g/dm^3^)
Sourness	Citric acid	77-92-9	0.28
Bitterness	Caffeine	58-08-2	0.195
Saltiness	Sodium chloride	7647-14-5	1.19
Sweetness	Saccharose	57-50-1	5.76

The pupils received one sample of each taste to familiarize themselves with the solutions, then they received a series of nine samples of which there were two samples representing each of the tastes being assessed, and one neutral sample containing water. All samples were served in disposable cups, (capacity 50 cm^3^) marked with unique, randomly assigned three-digit codes. During the evaluation, the participants took a mouthful of the solution (about 15 cm^3^), assessing each solution without rushing (at intervals of about 30 s). After each sample was tried, the participants rinsed their mouths with water supplied for this purpose, identical to the water used in the dilutions. The samples and the water were of the same temperature of about 20° C throughout the tests. The results were recorded on individual evaluation cards. The analysis of the results included the numbers (0; 1; 2) of the sweetness samples correctly identified by the evaluator.

### Analysis of yogurt selection preferences

Yogurt selection preferences were assessed using the principles of the scheduling method, according to the ISO 8587 sensory analysis methodology (ISO, 2006). Each participant was given 3 coded yogurt samples with the question: Which of the presented samples do you like best? As part of the subjective assessment, the participant could assess according to his/her criteria, taking into account any distinguishing features of the sensory quality. A plain unsweetened yogurt (A) and 2 raspberry-flavored yogurts were presented for evaluation: one raspberry yogurt was prepared *in situ* from plain unsweetened yogurt and frozen raspberries (B) and the other was a ready-made raspberry-flavored yogurt (C). All yogurt samples were from the same producer. The amount of fruit in the B yogurt was 9%, identical to what was stated by the C yogurt producer as for fruit content.

### Statistical analysis

Statistical calculations were made using the MS Excel spreadsheet, MS Office 2013, the R 3.1.2 statistical package under the GNU GPL license, and the STATISTICA 13 program, Stat Soft Polska.

Measurable data were characterized using the mean X and standard deviation S. The compatibility of the distribution of variables with the normal distribution was verified by the Shapiro–Wilk test. The significance of mean differences in the studied groups was checked using the t-test or ANOVA for many groups, while in the case of skewed distributions, their compliance in the groups was tested using the Mann–Whitney *U*-test or the Kruskal–Wallis test.

Percentage notation was used for nominal data. The differences between the percentages were assessed using a percentage difference significance test. The occurrence of relationships between nominal variables was verified with the *χ*^2^ test or Fisher’s exact test for tables *n* × *m* (*n*, *m* ≥ 2) in the case of small numbers. The correlation was assessed using the Cramer V-correlation coefficient for nominal data, and Gamma for ordinal data with an appropriate significance test.

In the multivariate analysis, ordinal multinomial logistic regression models were used for ordinal variables as the dependent variable. The significance of partial regression coefficients was estimated using the Wald test and the values of the odds ratio together with 95% confidence intervals. The assessment of the aggregate significance of the variables in the model was checked with the Wald test and the maximum likelihood-ratio test. The fit of the model was assessed with the information criteria AIC and BIC (lower values guaranteed a better fit of the model and at the same time its simplest form). In addition, multiple regression models were used for measurable variables as the dependent variable.

Statistical significance was determined at the level of *p* < 0.05.

## Results

Complete research material was collected from 160 out of 200 pupils – 70 (43.8%) girls and 90 (56.3%) boys (*p* = 0.12; NS). The age of the respondents ranged from 7 to 9 years with 31 seven-year-olds 31 (19.4%), 89 eight-year-olds (55.6%), 40 nine-year-olds (25.0%; *p* < 0.05).

The crude values of the body mass index (BMI) in the studied groups ranged from 11.3 to 24.58 kg/m^2^, while the standardized values from - 3.56 to 3.19. There were no significant differences in standardized BMI values based on whether the participant was male or female in the studied age groups (Table 2). The crude values of the percentage of adipose tissue in the studied groups ranged from 14.4 to 35.1%. Significantly higher values of the crude FATP index were recorded in the 8-year-old age group among girls (Table 2). There is a strong correlation between body weight assessments according to the BMI and FATP criteria (Table 2). It should be noted, however, that for 15% of all respondents, the percentage of adipose tissue (FATP) indicated a higher level of body mass assessment compared to BMI.

**Table 2:** Average values of the Body Mass Index (BMI) and the percentage of adipose tissue (FATP) in age groups, according to sex. Summary of body weight assessments according to BMI and FATP scales.

BMI (kg/m^2^)	Crude (*y*)	Total X ± S	Sex		*p*-value
	Age-standardized (*z*)		Girl X ± S	Boy X ± S	
Age	7	16.0 ± 2.4	15.6 ± 2.9	16.4 ± 1.9	*p* = 0.2; NS
		0.1 ± 1.3	−0.2 ± 1.4	0.4 ± 1.1	
	8	16.5 ± 2.5	16.4 ± 2.3	16.5 ± 2.7	*p* = 0.82; NS
		0.2 ± 1.4	0.2 ± 1.1	0.1 ± 1.6	
	9	17.1 ± 2.6	16.2 ± 2.7	17.5 ± 2.5	*p* = 0.11; NS
		0.3 ± 1.3	−0.2 ± 1.4	0.6 ± 1.2	
Total	Age-standardized	0.2 ± 1.3	0.1 ± 1.2	0.3 ± 1.4	*p* = 0.25; NS
FATP (%)	Crude (*y*)	Total X ± S	Girl X ± S	Boy X ± S	*p*-value
Age	7	20.6 ± 4.1	21.3 ± 4.4	19.9 ± 3.8	*p* = 0.38
	8	22.0 ± 4.8	23.1 ± 4.3	21.0 ± 5.1	*p* < 0.05
	9	21.8 ± 4.2	21.4 ± 3.5	22.0 ± 4.5	*p* = 0.69
BMI acc. WHO scale	Total *n* (%)	FATP acc. Tanita scale			Gamma correlation coefficient *p*-value
		Norm *n* (%)	Overweight *n* (%)	Obesity *n* (%)	
Underweight	6 (3.8)	6 (3.8)	0 (0.0)	0 (0.0)	0.95; *p* < 0.0001
Norm	108 (67.5)	94 (58.8)	13 (8.1)	1 (0.6)	
Overweight	30 (18.8)	3 (1.9)	19 (11.9)	8 (5.0)	
Obesity	16 (10.0)	0 (0.0)	6 (3.8)	10 (6.3)	
Total	160 (100.0)	103 (64.4)	38 (23.8)	19 (11.9)	

**Figure 1:**
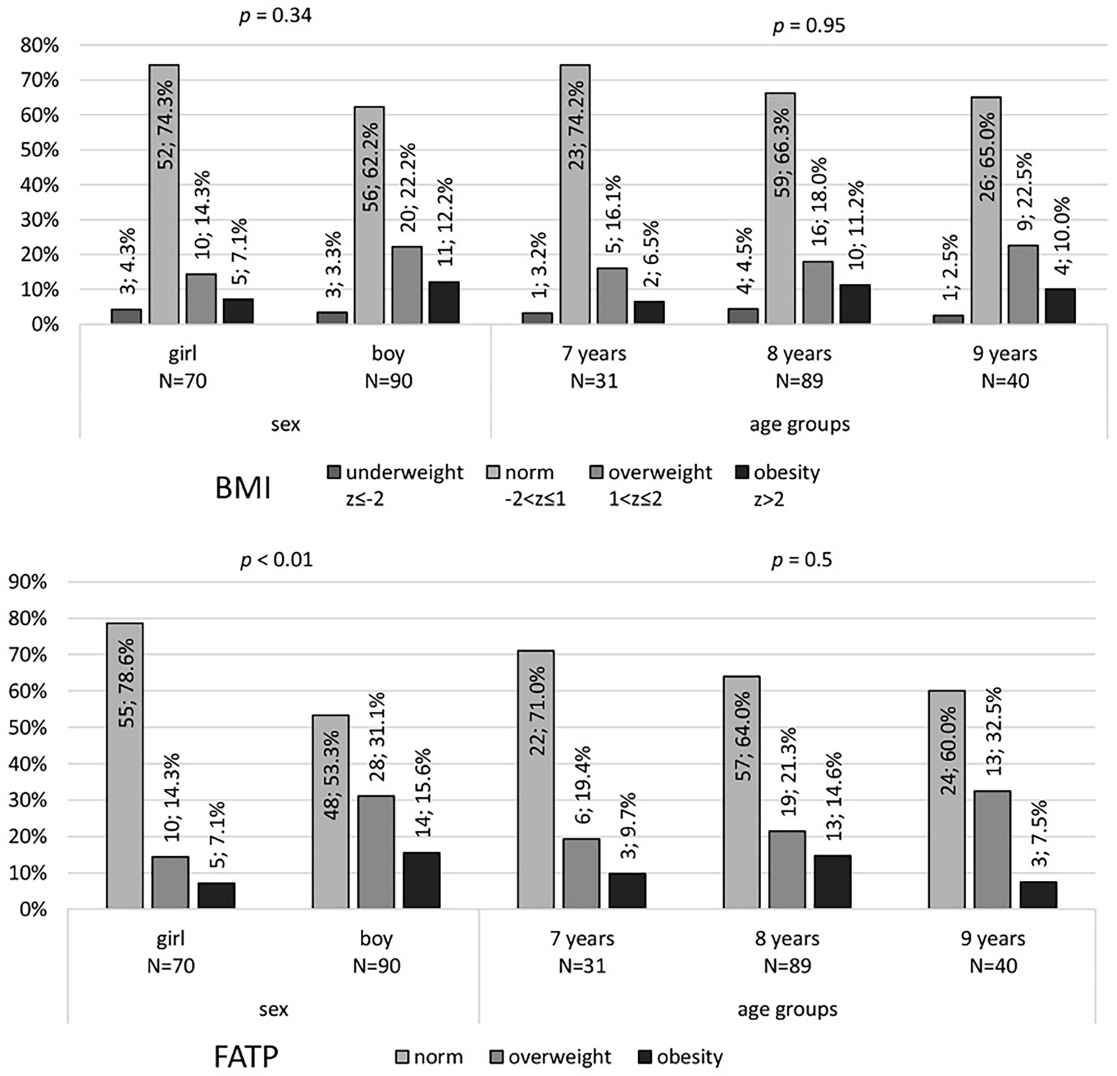
Assessment of the percentage of Body Mass Index (BMI acc. WHO standardized scale) and body fat (FATP acc. Tanita scale) including BioData. Data presented as a number of children n; percentage %.

The normative body weight according to the BMI value concerned almost 70% of the examined children (*n* = 108; 67.5%), while overweight (*n* = 30; 18.8%) or obesity (*n* = 16; 10%) – in total over 25%, and underweight only 3.8% (*n* = 6) of the examined group. Disorders (overweight, obesity) were more common in boys than in girls. Underweight was most common among children aged 8 years. Overweight and obesity most often occurred in children aged 9 years. These differences are not statistically significant – Figure 1. According to the FATP index, the normative body weight concerned about 65% of the examined children (*n* = 103; 64.4%), while overweight (*n* = 38; 23.8%) or obesity (*n* = 19; 11.9%) - over 30% of the studied group. The disorders (overweight, obesity) were significantly more frequent in boys than in girls (*p* < 0.01). Overweight and obesity were most common among children aged 9 years – Figure 1.

The study on the frequency of fermented milk product consumption did not reveal any significant differences in terms of sex, BMI, and FATP (Table 3).

**Table 3:** The frequency of fermented milk product consumption, taking into account sex, the BMI category and the percentage of body fat (FATP).

Group		Total *N* (100%)	Sour milk products consumption (yogurt, kefir, buttermilk)					*p*-value
			Every day, 1 cup *n* (%)	3–4 cups a week *n* (%)	A few cups a month *n* (%)	Occa-sionnally *n* (%)	Never *n* (%)	
Sex	Girl	70	15 (21.4)	23 (32.9)	15 (21.4)	12 (17.1)	5 (7.1)	*p* = 0.88; NS
	Boy	90	20 (22.2)	26 (28.9)	16 (17.8)	19 (21.1)	9 (10)	
BMI	Underweight	6	2 (33.3)	0 (0)	1 (16.7)	1 (16.7)	2 (33.3)	*p* = 0.88; NS
	Norm	108	24 (22.2)	33 (30.6)	21 (19.4)	23 (21.3)	7 (6.5)	
	Overweight	30	5 (16.7)	10 (33.3)	6 (20.0)	5 (16.7)	4 (13.3)	
	Obesity	16	4 (25.0)	6 (37.5)	3 (18.8)	2 (12.5)	1 (6.3)	
FATP	Norm	103	23 (22.3)	29 (28.2)	20 (19.4)	20 (19.4)	11 (10.7)	*p* = 0.34; NS
	Overweight	38	9 (23.7)	9 (23.7)	8 (21.0)	10 (26.3)	2 (5.3)	
	Obesity	19	3 (15.8)	11 (57.9)	3 (15.8)	1 (5.3)	1 (5.3)	
Total		160	35 (21.9)	49 (30.6)	31 (19.4)	31 (19.4)	14 (8.8)	-

Table 4 summarizes the results of the sweetness recognition test and yogurt preference taking into account the sex, BMI, and FATP of the respondents.

**Table 4:** Results of the sweetness recognition test and yoghurt preference, taking into account the sex, BMI and FATP of the respondents.

Group		Total *N* (100%)	Recognition of sweetness			Yogurt preference		
			0 *n* (%)	1 *n* (%)	2 *n* (%)	A *n* (%)	B *n* (%)	C *n* (%)
Sex	Girl	70	10 (14.3)	20 (28.6)	40 (57.1)	5 (7.1)	18 (25.7)	47 (67.1)
	Boy	90	7 (7.8)	42 (46.7)	41 (45.6)	20 (22.2)	20 (22.2)	50 (55.6)
	*p*-value		*p* = 0.05 NS			*p* = 0.03; *p* < 0.05		
BMI	Underweight	6	0 (0)	2 (33.3)	4 (66.7)	1 (16.7)	1 (16.7)	4 (66.7)
	Norm	108	4 (3.7)	43 (39.8)	61 (56.5)	19 (17.6)	26 (24.1)	63 (58.3)
	Overweight	30	8 (26.7)	10 (33.3)	12 (40.0)	4 (13.3)	6 (20.0)	20 (66.7)
	Obesity	16	5 (31.3)	7 (43.8)	4 (25.0)	1 (6.3)	5 (31.3)	10 (62.5)
	*p*-value		*p* < 0.0001			*p* = 0.86; NS		
FATP	Norm	103	3 (2.9)	36 (35.0)	64 (62.1)	20 (19.4)	25 (24.3)	58 (56.3)
	Overweight	38	6 (15.8)	17 (44.7)	15 (39.5)	4 (10.5)	9 (23.7)	25 (65.8)
	Obesity	19	8 (42.1)	9 (47.4)	2 (10.5)	1 (5.3)	4 (21.1)	14 (73.7)
	*p*-value		*p* < 0.0001			*p* = 0.35; NS		
Total		160	17 (10.6)	62 (38.8)	81 (50.6)	25 (15.6)	38 (23.8)	97 (60.6)

Relatively high efficiency in identifying the sweetness was obtained. 50.6% of the panelists recognized the samples of this taste without fail. A higher percentage of girls than boys is noticeable here. 57.1% versus 45.6% respectively. The sex of the participant was observed as having a significant influence on yogurt preference; where girls significantly more often preferred the ready-made raspberry yogurt sample (C) - 67.1% compared to 55.6% of boys. On the other hand. Boys significantly more often (22.2%) than girls (7.1%) indicated a preference for the sample of plain unsweetened yogurt (A). A significantly higher recognition of sweetness was observed among underweight children. A significantly higher recognition of sweetness was observed among children with normal body weight (Table 4).

**Figure 2:**
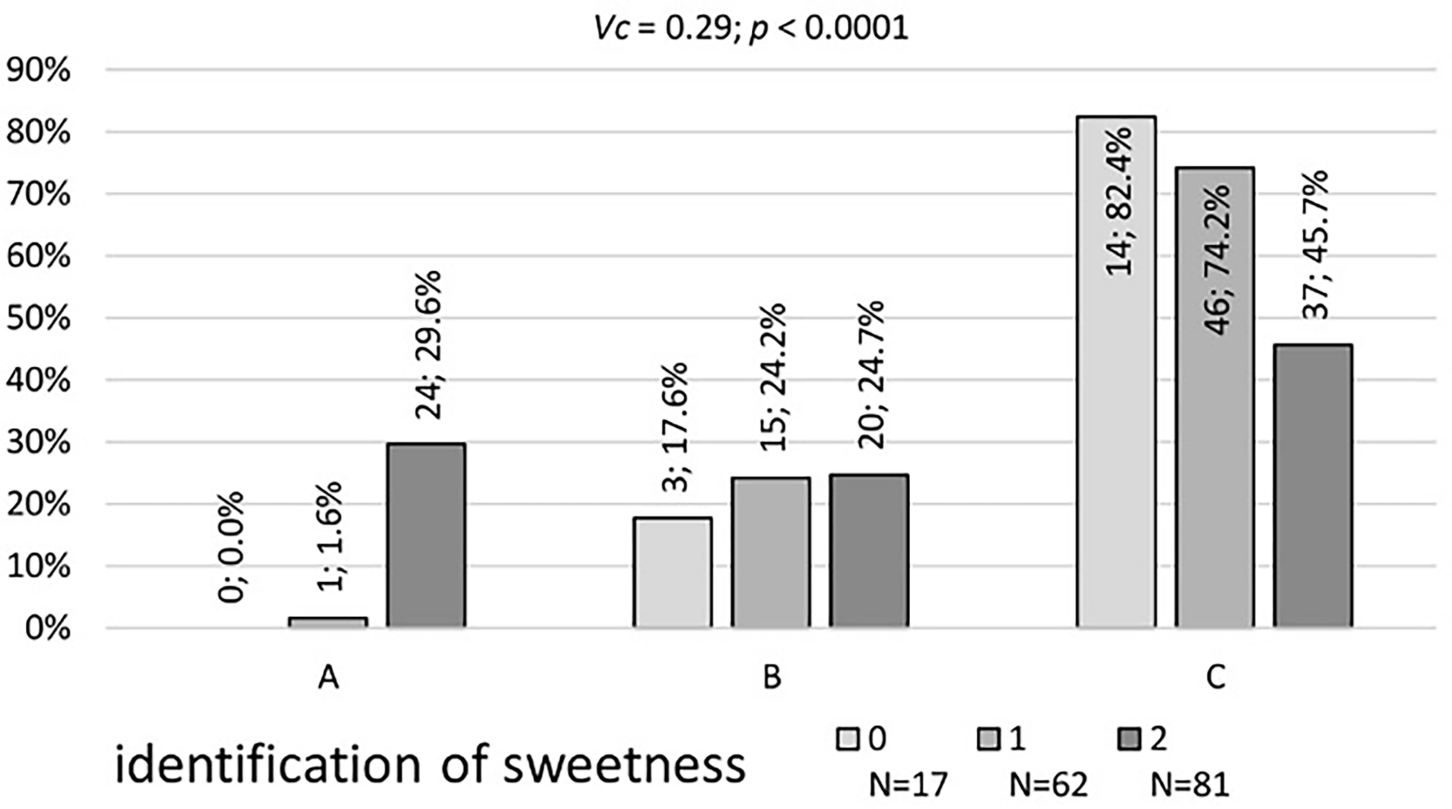
The level of correlation (V Cramer correlation coefficient) between the results of the yoghurt preference test **(A–C)** and the sweetness recognition test (0, 1, 2).

Table 5 presents the results of fermented milk product consumption frequency and the ability to identify sweetness by the respondents. No significant correlation was found between the frequency of consumption of fermented milk products and the results of the sweetness identification test (Table 5). Significant differences were observed in the preferences of yogurts about the identification of sweetness (Figure 2).

**Table 5:** Results of the sweetness recognition test taking into account the frequency of fermented milk product consumption.

Sour milk products consumption (yogurt, kefir, buttermilk)	Total *n* (100%)	Recognition of sweetness			*p*-value
		0 *n* (%)	1 *n* (%)	2 *n* (%)	
Every day 1 cup	35	6 (17.1)	16 (45.7)	13 (37.1)	*p* = 0.09; NS
3–4 cups a week	49	6 (12.2)	22 (44.9)	21 (42.9)	
A few cups a month	31	4 (12.9)	11 (35.5)	16 (51.6)	
Occasionally	31	0 (0.0)	10 (32.3)	21 (67.7)	
Never	14	1 (7.1)	3 (21.4)	10 (71.4)	
Total	160	17 (10.6)	62 (38.8)	81 (50.6)	-

Among the predictive factors taken into account in the full logistic regression model. BMI has a significant impact on the change in the level of sweetness identification. The estimated probability of increasing the level of sweetness identification is statistically significantly lower in obese children (0.13 times lower than in overweight children; Table 6).

**Table 6:** Estimation of the ordered logistic regression model parameters for the identification of sweetness (0.1.2) depending on BioData. frequency of fermented milk product consumption, and yogurt preferences.

Predictive factor	Regression coefficient	Standard error of the coefficient	*p*-value	Odds ratio	Confidence interval of 95%	
	*β*_i_	*B*_std_ *β*_i_		*e* ^βi^		
BMI	Reference group: underweight					
Norm	−0.44	0.87	0.61	0.64	0.09	3.20
Overweight	−1.49	0.93	0.11	0.23	0.03	1.27
Obesity	−2.08	0.99	0.04	0.13	0.01	0.79
Model	Wald test		Maximum likelihood test		AIC	BIC
	*χ* ^2^	*p*-value	*χ* ^2^	*p*-value		
	13.9	*p* = 0.003	14.5	0.0002	299.5	314.9

## Discussion

Yogurt is traditionally produced using *Lactobacillus delbrueckii* ssp. *bulgaricus* and *Streptococcus thermophilus bacteria*, which produce lactic acid (Zhang et al., 2020). In the industrial production of yogurt, cow’s milk with various amounts of fat is most often used, moreover, other ingredients may be added, including skim milk, powdered milk, whey protein, lactose, sweeteners, i.e., glucose, saccharose, aspartame, and sucralose, fruit, artificial and natural dyes and flavors (Tremblay and Panahi, 2017). The current consumption of added sugars consumed by children significantly exceeds the recommended dietary intake (Mennella et al., 2014).

The results of this analysis indicate the significance of the childhood overweight and obesity issue. The obtained data on the level of BMI and body fat content to some extent coincide with the results of other researchers. A study conducted on over 12,000 American children aged 6–18 confirms the problem of excess body weight in children. About 30% of boys and girls were overweight or obese, and about 15% were obese. On average, adipose tissue accounted for 17.6% of boys’ body weight and 23.9% of girls’ body weight. Our results indicate the identification of 28.8% of children with excess body weight and 10% of children who are obese. Boys were more frequently affected by weight disorders, which was not confirmed in American studies. Moreover, the average content of adipose tissue in our study was 21.1 and 22.6% in boys and girls, respectively. The differences in the content of adipose tissue in the authors’ study and American studies probably result from the growth spurt that occurs at this age (Going et al., 2011). However, in another study involving over 850 children aged 6–10 from Saudi Arabia, the obtained results differ from the previously mentioned results. Children with BMI classified as overweight or obese constituted 22.3% of all respondents, of which 9.9% were obese children. Moreover, the overall prevalence of overweight or obesity was significantly higher among girls, which contradicts the results presented in this study. According to the percentage of adipose tissue, the total incidence of overweight and obesity in the study from Saudi Arabia the result was 7% in boys and 6.6% in girls, while our results indicate that as many as 46.7% of boys and 21.4% of girls are overweight or obese. In both studies, there is a correlation between the assessment of body weight according to the BMI criterion and the adipose tissue content, although, in contrast to our studies, the incidence of overweight or obesity in the Saudia Arabian study was higher using BMI than the percentage of adipose tissue (Al-Mohaimeed et al., 2015).

The frequency of fermented milk product consumption in our study did not depend on gender, BMI, and body fat content. Over 30% of the respondents consumed 3–4 cups of fermented milk products a week, while 8.8% of children did not eat this type of food. A study of more than 5,000 American children aged 2–18 confirmed that there was no gender-related connection with yogurt consumption. Children participating in the study were divided into two groups, frequent and rare yogurt consumers, and there was no correlation between the frequency of yogurt consumption and body weight (Zhu et al., 2015). Another study of over 3,700 participants aged 8–18 showed different results. The respondents were classified as consumers of dairy products and persons not consuming such products, and persons consuming and not consuming yogurts. The study showed that only 30% of the respondents consumed less than one serving of dairy products a day, our results indicate that less than one serving a day of fermented dairy products are consumed by 78.1% of children, milk consumption was not included in this, which probably contributes to such a large discrepancy in the results. Yogurt consumers were associated with a lower incidence of overweight and obesity, a smaller waist circumference, and a lower sub-scapular skin fold, compared to those who did not eat yogurt. On the other hand, dairy consumers have only been associated with a lower thickness of the subscapular skin fold (Keast et al., 2015).

An important issue affecting the overall consumption of sugar is the preference for sweetened or sweet products. Ready-made fruit yogurts are characterized by the sweetness which is made by adding sugar or other sweeteners. Our own study showed that girls more often chose the sample of ready-made yogurt as the most preferred, while boys significantly more often indicated their preference for plain unsweetened yogurt. Another study, aimed at analyzing the etiology of overweight and obesity, used surveys that were conducted in Italy, Estonia, Belgium, Sweden, Germany, Spain, Cyprus, and Hungary. The taste preferences of 1705 children aged 6–9 years were analyzed. Among other things, the taste preferences of different amounts of sucrose content in apple juice were investigated. Most of the children preferred the sample with the addition of sucrose, and the age of the respondents was associated with a greater likelihood of preferring sweets. Gender did not influence the preference for sweetness (Lanfer et al., 2013).

In our study, significantly higher recognition of sweet taste was observed in children with BMI classified as underweight and in children with normal body fat content. This relationship is not confirmed by the studies by Mennell et al., Involving 108 subjects aged 5–10 years. About 30% of the children participating in the study had a BMI indicating overweight or obesity, which is consistent with our results. However, the sweet taste preference was related to the height of the subjects, and no correlation with the percentage of body fat was found. There were no differences in preferences related to age (Mennella et al., 2014).

The sweetness threshold is an important issue in terms of food preferences and sugar consumption in the diet. A study in children aged 7–14 years looked at the difference in the sucrose detection thresholds. 44% of the examined children had a BMI indicating overweight or obesity. The analysis showed that older children were more sensitive to sweetness, and the girls had lower detection thresholds than the boys. There was no correlation between the detectability of sucrose and the BMI index, however, a correlation was noted that the higher the percentage of adipose tissue, the lower the sucrose detection thresholds (Joseph et al., 2016).

Research conducted by Ashi et al. investigated the perception of sweet taste about BMI in 13-to 15-year-old schoolchildren from three different countries (Italy, Mexico, and Saudi Arabia). A total of 669 subjects were studied with 20% being obese and 16.3% overweight. A statistically significant difference was found regarding BMI among children from the three countries, with the highest average found among Saudi children, followed by Mexican and Italian children. A statistically significant difference regarding sweet taste threshold when comparing the BMI groups was discovered only for children from Saudi Arabia. However, no correlation was found between BMI and sweet taste recognition threshold (Ashi et al., 2019). Another study evaluated the relationship between obesity with a liking for fatty and sugary foods as well as fruits and vegetables. The study included 366 children aged 7–9 years, and assessed BMI waist-SD ratio and body fat mass index. The study showed that vegetables were preferred to me by obese children, while there was no difference in fruit preference. Boys were more likely to prefer sugar-containing products than girls. Interestingly, no relationship was found between preference for any of the food categories and obesity (Hill et al., 2009). A study by Overberg et al. analyzed the taste sensitivity of obese and normal-weight children and adolescents. The study compared the taste sensitivity of 193 subjects aged 6–18 years. Obese subjects were found to have a significantly lower ability to identify the right taste qualities about the total score. In addition, the female gender had a better ability to identify the taste. When evaluating the intensity of sweet taste, obese children rated the intensity of three of the four concentrations significantly lower (Overberg et al., 2012). The study by Park et al. aimed to examine differences in taste detection thresholds between normal-weight and obese adults. Forty-one adults in their twenties participated in the study. The study found that the obese group had higher recognition thresholds for sweet, salty, sour, and bitter tastes although the ist difference was only for salty tastes. Smoking affected taste recognition, with smokers having higher thresholds than non-smokers (Park et al., 2015).

The strengths of this study include a broad comparison of the ability to recognize sweetness to numerous other variables, both in terms of yogurt choice preferences, as well as the frequency of consumption and anthropometric measurements. According to the current state of knowledge, the topics covered in the research were not analyzed in other works. In addition, all results were obtained by the standards and guidelines specified for each stage of the study. This analysis also has several limitations that require further development or clarification. The analysis of the frequency of consumption of fermented milk products should in the future be expanded to include questions about a single, specific product and clarify the issue related to fat content and possible sweetening of these products. Moreover, extending the study to other food products or, going further, an analysis of the daily diet, would allow for more precise conclusions. An interesting solution seems to be the use of a 24-h interview or the current recording method. The results obtained in this way would be more precise and would cover the quantitative and qualitative aspects of the consumed products. The level of physical activity and its effect on body weight is a very well-described issue. In the continuation of research on this topic, it is also worth considering this aspect. An interesting additional issue is also the determination of the sucrose detection threshold in aqueous solution and yogurts, as the results may vary depending on the medium used. The topics discussed herein require further analysis.

## Conclusion

Based on the results obtained were found, a significant number of children aged 7–9 were overweight or obese about BMI and FATP categories. These conditions were more common in 9-year-olds than in younger children, especially in boys. There was no significant relationship between the body mass of the subjects when considering both BMI and FATP and the frequency of fermented milk product consumption. A correlation was found between the values of body mass indexes and the recognition of sweetness, which was significantly better recognized by children who were underweight (according to BMI) and those within the body weight norm (according to FATP). No significant relationships were found between the frequency of fermented dairy product consumption and the result of the sweetness identification test, while a significant differentiation was observed in yogurt preference including the identification of sweetness - subjects with a higher ability to recognize sweetness significantly more often preferred plain unsweetened yogurt. The results of the present study do not answer all the questions and do not fully explain the observed relationships, so it should be continued. In further research plans, it would also be worthwhile to consider a broader range of foods (e.g., vegetables) in the context of several sensory attributes (e.g., bitter taste, odor, and color), which are among the important elements that influence food evaluation, shape food preferences and habits, and may contribute to the maintenance of normal body weight.

## Data availability statement

The raw data supporting the conclusions of this article will be made available by the authors, without undue reservation.

## Ethics statement

The studies involving human participants were reviewed and approved by Bioethics Committee of the Medical University of Silesia in Katowice. Written informed consent to participate in this study was provided by the participants’ legal guardian/next of kin.

## Author contributions

MK: conceptualization and supervision. MK, WS, AK, and AB: methodology. EN: software, validation, resources and data curation. MK and WS: formal analysis and writing—original draft preparation. MK, WS, AK, and AB: investigation. MK, WS, and EF: writing—review and editing. MK, WS, AK, AB, and EF: visualization. All authors contributed to the article and approved the submitted version.

## Funding

Research results were collected as part of an educational project addressed to second-grade primary school children (contract WND-POWR.03.01.00-00-C036/16 from the National Center for Research and Development).

## Conflict of interest

The authors declare that the research was conducted in the absence of any commercial or financial relationships that could be construed as a potential conflict of interest.

## Publisher’s note

All claims expressed in this article are solely those of the authors and do not necessarily represent those of their affiliated organizations, or those of the publisher, the editors and the reviewers. Any product that may be evaluated in this article, or claim that may be made by its manufacturer, is not guaranteed or endorsed by the publisher.
